# Assessment on Practicing Correct Body Posture and Determinant Analyses in a Large Population of a Metropolitan Area

**DOI:** 10.3390/bs13020144

**Published:** 2023-02-08

**Authors:** Paolo Montuori, Luigi Mauro Cennamo, Michele Sorrentino, Francesca Pennino, Bartolomeo Ferrante, Alfonso Nardo, Giovanni Mazzei, Sebastiano Grasso, Marco Salomone, Ugo Trama, Maria Triassi, Antonio Nardone

**Affiliations:** 1 Department of Public Health, University “Federico II”, Via Sergio Pansini n° 5, 80131 Naples, Italy; 2 General Directorate of Health, Campania Region, Centro Direzionale is. C3, 80143 Naples, Italy

**Keywords:** posture, ergonomics, multiple linear regression

## Abstract

An incorrect posture can generate stress of the spine and can be the cause of musculoskeletal disorders. Considering the extensive use of the computer, which worsens posture disorders, among workers, is important to analyze the phenomenon in order to reduce his impact on industry. The aim of this study is to assess determinants regarding posture in a large population of a metropolitan area. A total of 1177 questionnaires was analyzed. The majority of sample showed good knowledge and attitude regarding correct posture; most of the sample, 70.4% was aware of the definition of posture and 68.7% feel that not enough attention is paid at posture at workplace. Despite the good predisposition, only 2.8% of the sample consult a specialist for posture. The multiple linear regression analysis shows that those who have higher knowledge and best attitudes will consequently have good behaviors in maintaining a correct posture. Furthermore, age and education resulted main drivers of correct posture in any model considered. The results enlighten the necessity of conducting further studies to analyze attitudes of the general population and suggest improving educational and training programs to the enrichment of knowledge and to correct posture behaviors.

## 1. Introduction

An incorrect posture can generate stress of the spine and girdles (scapular and pelvic), and this can be the cause of musculoskeletal disorders perceived as pain [1,2,3]. The problem of posture is very important and can also affect schoolchildren and workers [4,5,6,7]. In 25–60% of children and adolescents, postural weakness can be the cause of the form of hanging shoulders, hollow back, or anti-verse pelvis [8,9,10]. Yet, an early evaluation of posture as long as a tailored training program may prevent future impairments [11].

The prevalence of postural dysfunctions is very difficult to measure because the factors that can influence posture are many and in the various studies the populations examined are not general but always different and, often, not very representative, such as in the case of the school population, employees and health care workers [12,13,14]. Nevertheless, the theme of correct posture finds its application in the school environment regarding pre-adolescent students in particular. A working group correlated the back pain of schoolchildren with an excessive weight of school backpacks, thus proposing a school curriculum that limits the weight of backpacks [7,15]. The problem of correct posture also becomes important considering the excessive use of the computer [6,16]. A review of the literature confirms the association between computer use and musculoskeletal disorders [16]. A survey of 1428 employees, using a questionnaire, showed the following prevalence rates of disorders at 12 months, distributed by body area: head/neck (42%), lower back (34%), upper back (28%), wrists/hands (20%), shoulders (16%), ankles/feet (13%), knees (12%), hips (6%) and elbows (5%) [6]. In a study of 2431 video display terminal workers, the six-monthly incidence of musculoskeletal symptoms was 18% for the shoulder–neck region, 14.8% for the lumbar region and 21.7% for the upper limb region [17].

Many studies that have evaluated correct posture have been carried using photographs, the photogrammetric method, inclinometers, electronic goniometers and radiography [18,19,20,21,22]. Convolutional neural networks were applied to realize an algorithm based on deep learning to develop smart chairs designed to improve posture in childhood [23]. In a case control study, the posture of two groups of subjects was studied using photographs of the sagittal planes in a sitting and standing position to determine the extent of the forward head position [22]. The photogrammetric method with a moiré effect on the Mora 4G CQ Elektronik device was used to examine the body posture of 101 children [20]. One study monitored the neck posture of crane operators during 2 h of work through inclinometers [18]. The use of electronic goniometers can also be used to study angular displacements in the lower back and neck [21]. Standardized lateral planar radiographs in a neutral standing position for the entire spine were used to study the relationships between forward head posture and lumbar–pelvic sagittal alignment in elderly people with lower back pain [19].

Other authors have used questionnaires to investigate on practicing correct body posture. Literature research has shown that behaviors are the result of knowledge, attitudes or how they interact with each other [24]. In fact, several studies—not linked to correct body posture—analyze practices according to knowledge and attitudes [25,26,27,28]. In particular, the advantages of applying the KAP survey model to the problem of posture are obvious. The KAP survey model allows for the evaluation of whether the knowledge of a population affects attitudes and how, in the end, these attitudes influence behaviors (understood as maintaining a correct posture or suffering from musculoskeletal disorders). Among all those who have used the questionnaires on practicing correct body posture, none analyze knowledge, attitudes and behaviors simultaneously, except one that analyses only behaviors (understood as practices) with knowledge and does not consider attitudes [29]. However, this study uses a small and specific population comprising 210 males and 134 females and desktop users of different professions. Alley et al., based on the theory of planned behaviors, studied trough questionnaires the knowledge about sitting posture, sedentary behaviors (understood as sitting time and physical activity) and the intentions to change behaviors in an Australian population composed of 494 seniors over the age of 65 [30]. Also, Epstein et al., analyzed, using questionnaires and quizzes, only the knowledge of correct posture in 457 students [31]. Finally, the association between behaviors, understood as bad postures, and back and neck pain in a small population of adolescents was analyzed by Meziat-Filho et al. [32]. To the best of our knowledge, no study has simultaneously analyzed knowledge, attitudes, and behaviors on practicing correct body posture in a large population of metropolitan area.

The main aim of this study is evaluating knowledge, attitudes and behaviors simultaneously on practicing correct body posture in a large metropolitan area to possibly propose health education programs targeted at promote a correct posture and conversely reduce the risks associated with maintaining an incorrect posture.

## 2. Material and Methods

### 2.1. Study Design and Sample Calculation

This cross-sectional study was conducted administering questionnaires to adults from the metropolitan city of Naples (Italy) with a population of 967,069. The study was conducted from the beginning of November 2021 to the end of March 2022. Using a snowballing sampling method among university, working places and community centres, a total of 1670 subjects were selected to participate at the study. Among those, 1177 accepted to participate and returned the questionnaire, with a response rate of 70.5%. The criteria of inclusion in the study were that participants were aged 18 and older and residing in the metropolitan area of Naples. The required sample size was calculated using Slovin’s formula with the objective of obtaining a representative sample within a margin of error of 3%, and a confidence interval of 95%, determining a final number of subject to be recruited of 1523. Finally, after accounting for a 30% nonresponse rate, the estimated total desired sample size became 1066; nonetheless, we received slightly more questionnaires than expected.

### 2.2. Procedures

During the study period, experienced interviewers submitted the questionnaire to participants from Monday to Friday between 10:00 a.m. and 8:00 p.m. to avoid oversampling nonworking individuals. The interviewers, at the beginning of the submission, stated that they were conducting a study on behalf of the University of of Naples “Federico II”, providing information to the participants about the nature and scope of the research, the methodology, that their participation was on a voluntary basis, that all the collected information would be processed anonymously and confidentially, and that they could end their participation at any time without disclosing a reason. Verbal informed consent was obtained prior to progressing with the interview. No incentive for participation or survey completion was provided. The present study was carried out in conformity with the Declaration of Helsinki and ethical clearance was obtained according to local legislation.

### 2.3. Data Collection

The questionnaire used consists of a first part containing basic information about the participants (age, gender, level of education, profession, smoking) and 3 groups of questions on knowledge, attitudes and behaviors relating to correct posture, for a total thirty-six questions. The inclusion or exclusion of questions about information, attitudes and behaviors was conducted as suggested by the KAP survey model. The steps to develop the KAP survey model were as follows:

(1) Construction of the survey protocol;
(2) Elaboration of the questionnaire;
(3) Conducting of the field survey;
(4) Analysis and interpretation of the data.

To elaborate the questionnaire, questions were initially formulated to support the “Study Objectives” (to formulate the questions, in accordance with the KAP survey model, knowledge was elaborated as a set of understandings, attitudes as ways of being, behaviors such as actions or attunements). Subsequently, the questions were reduced by removing those that require excessive information. Once the previous step had been performed, the more complex questions or questions not in line with the model were modified/removed. Knowledge and attitudes were measured using a three-point Likert scale with choices for “agree”, “uncertain” and “disagree”, while behavioral questions were formulated with four answers: “never”, “sometimes”, “often” and “always”. In parallel, a pilot study was conducted to verify the questionary and verify the reliability of the questions. Finally, all the questionnaires collected were digitized by inserting the results in an Excel worksheet (MS Office, 2019). After a numerical decoding of the answers, the Excel worksheet was statistically analyzed using IBM SPSS Statistics 27 software.

### 2.4. Statistical Analysis

The analysis was applied in 2 stages. In the first phase, the determinants of the population were analyzed, understood as age, gender, smoking habit, sentimental status, having children and how many children. In the second step, multiple regression analysis (MLRA) was performed, a mathematical technique that analyzes how many independent variables affect a dependent variable. The goal of multiple regression analysis (MLRA) is therefore to analyze the linear relationship between independent and dependent variables.

The main results of the MLRA consider the statistical significance applied to the regression model (*p*-value < 0.05), the estimate and statistical significance applied to the beta coefficients (*p*-value < 0.05) and the constant of determination (R-squared and R-squared corrected), which is used to measure how much variation in the result can be determined from the variation of the independent variables. Three MLRAs were developed, as well as variables undoubtedly linked to subsequent results of interest:

(1) Knowledge of correct posture (Model I);
(2) Attitudes about correct posture (Model II);
(3) Behaviors related to correct posture (Model III).

The dependent variables (knowledge, attitudes and behaviors) were obtained by summing up the scores within the corresponding questions ranging from 12 to 36 for knowledge and attitude, and therefore considered as total knowledge (KTOT) and total attitude (ATOT), and from 15 to 60 for behaviors and therefore considered as total behaviors (BTOT). The independent variables were enclosed in the overall models: gender (1 = male, 2 = female); age in years; smokers (1 = smoker, 2 = nonsmoker); sentimental status (1 = single, 2 = not single); having children (1 = having children, 2 = not having children); if they had children, they were asked how many children they had. In model I, we analyzed knowledge with respect to independent variables; in model II we analyzed attitudes with respect to independent variables including knowledge among independent variables; while in model III we analyzed behavior with respect to independent variables, also including knowledge and attitudes between independent variables. In the analysis, we considered attitudes and knowledge as indices rather than as a scale, meaning that each observed variable (A1,…, A12 and K1,…, K12) is assumed to cause the associated latent variables (attitude and knowledge). The relationship between observed and latent variables is formative. Therefore, no correlations are required between the observed variables. Conversely, the relationship between the observed variables (B1,…, B15) and the latent variable behavior could be considered reflexive (Cronbach’s alpha = 0.825). All statistical tests were two-sided, and results were considered statistically significant if *p*-values were less than or equal to 0.05.

## 3. Results and Discussions

A total of 1670 questionnaires were distributed; out of those, 1177 were returned (equal to 70.5% of the total). The study of the frequencies of the characteristics of the population (Table 1) showed that 50.8% of the respondents were males while 49.2% were females; this pattern of distribution confirms that the sample is homogeneous. The mean age of respondents was 37.57; the minimum age was 18 years (age less than 18 years was considered as an exclusion criterion), while the maximum age was 70. Regarding the habit of smoking, 72.5% said they did not smoke. Respondents declared that their sentimental status was, for a total of 66.4%, “in a relationship” while 55.9% of the study population said they did not have children.

Table 2 includes response rates for knowledge-related questions. In particular, the sample answered the questions related to the definition of posture correctly (K1 70.4%; K2 54.5%; K3 48.7%). Many of the respondents believe it is necessary to correct a bad posture (71.3%) and in this regard a high percentage of respondents know the main treatments to improve posture (K5 81.1%; K6 73.4%; K7 80.8%). Regarding the importance of knowledge on ergonomics to promote a good posture, the result is preoccupant, in fact the sample responded incorrectly at question K10 in 38.7% of the cases. This result already indicates to direct a health education program targeted at increasing knowledge in accordance with Ketola et al., which evidenced that musculoskeletal disorders are reduced in a group undergoing ergonomics training [33]. Finally, answering question K11, 36.7% of the sample was uncertain or responded incorrectly about the correct posture to assume when using a computer. This clearly shows that some important notions about posture are not sufficiently spread in the population, considering that is fully accepted that maintaining posture when using computer is necessary to reduce risk of musculoskeletal disorders [34,35].

Table 3 shows the percentages of response to attitude questions. Specifically, 62.8% of respondents believe that the topic of posture is not adequately treated by the mass media as well as the correct posture in the workplace (68.7%). In addition, 78.6% of the subjects in the study consider it difficult to maintain a correct posture for too long, preferring behaviors not characterized by maintaining an incorrect posture (41.4% of affirmative answers to question A6). Regarding question A9, 81.1% of the study population believe that children’s school backpacks should have weight limits. The study population, therefore, appears to be worried about the issue of the excessive weight of backpacks in children and adolescents. In fact, Shamsoddini et al., in 2010, found that excessive weight of backpacks in secondary school students is associated with an increase in musculoskeletal discomfort with a different incidence in different body locations [36]; in particular, a frequency of 38.1% was measured for shoulder disorders, 27.6% for neck disorders and 16.7% for back disorders. Recently, another study group noted that there is a correlation between the excessive weight of the backpacks of elementary school students and the onset of back pain [7]. Unfortunately, to the best of our knowledge, there are no previous studies regarding attitudes toward posture present in the literature to compare with; however, these results show a good attitude towards maintain a correct posture in the study population.

Regarding behavior, the frequencies of responses to this section have been set out in Table 4. A high percentage of respondents do not practice postural gymnastics (79.6%) nor turn to a posturologist for joint problems (81.9%). Of the total, 45.5% said that they “sometimes” maintain a correct posture when using the computer; on the other hand, 47.2% “never” maintain a correct posture when watching TV. This data is interesting because it led the deduction that the use of a computer, perhaps in the workplace, is perceived as a moment in which it is necessary to try to maintain a correct posture. However, very few use a riser to improve computer posture (12.7%), even fewer use a footrest (3.3%) and 46.4% of the subjects participating in the study said they sit for more than 4 h during the day. It is interesting to note that 49.2% of respondents said they had never suffered from sciatica, while 40.6% “sometimes” suffered from back pain and 34.4% “sometimes” suffered from neck pain. Also, in this case, it can be deduced that many hours a day of sitting posture led to a higher incidence of musculoskeletal problems in accordance with Daneshmandi et al.’s finding that excessive sitting behavior is a risk factor for many adverse health outcomes [37].

In a multiple linear regression, no statistically significant correlation (*p* > 0.05) was encountered between knowledge and sex, sentimental status and having or not having children while knowledge was statistically associated with age, smoking habit, number of children and educational qualification (Model I, Table 5). This study evidenced that younger subjects and those who have a low number of children have higher knowledge. Unfortunately, no previous studies have examined the level of knowledge about correct posture in the general population at different ages to compare with; but, only in specific age ranges, such as in Alley et al., who analyzed knowledge about correct posture in a population aged over 65, found a good level of knowledge associated with the health risks linked to posture [30]. Also, Epstein enlightened the importance of knowledge about ergonomics, but only exclusively in adolescents [31]. Regarding the level of education, a positive correlation between level of education and degree of knowledge was found. Another result was that not having smoking habits is positively correlated to a higher level of knowledge on posture. This result allows us to assert that in the study population, both young people and those who do not smoke have a higher level of knowledge, and therefore are more aware of the problem of posture.

A statistically significant correlation (*p* < 0.05) between attitude and age, smoking habit, sentimental status, presence of children, number of children, education and knowledge was found (Model II, Table 5). Younger subjects have better attitudes toward correct posture, as well as nonsmokers. Also, those in a sentimental relationship (no single) have positive attitudes toward correct posture, as well as those who have children. In addition, there is a positive attitude toward correct posture that increases with an increase in number of children. Moreover, in Model II, a negative attitude toward correct posture with higher educational level emerged while in Model I, a higher educational level is statistically significant with higher knowledge; therefore, these results—although not comparable due to the lack of other similar studies—may lead to the hypothesis that the educational level interacts with attitudes to correct posture through knowledge. In fact, the educational level affects the level of knowledge on posture but not attitudes, while knowledge is positively correlated with attitudes. No statistically significant correlation between attitudes and gender was encountered.

In Model III, Table 5, a statistically significant correlation was observed between behaviors and age, sentimental status, number of children, education, knowledge, and attitudes while there is no significant correlation between behaviors and sex, smoking habit and having or not having children. The first evidence of this model shows that younger subjects have better behaviors in maintaining posture; this result disagrees with the only similar study carried out by Nejati et al., in which no correlations were identified between incorrect behaviors and age of the subjects studied [38]. Regarding the sentimental status, the correlation is positive, so being in a relationship is a condition that influences behaviors to maintain a correct posture. Oddly, in Model III, no significant correlation between behaviors and having or not having children was observed, while people who have more children have a negative behavior in maintaining a correct posture. Although not comparable due to the lack of other similar studies, these results highlight a relevant problem, that is, subjects who have more children not caring about maintaining a correct posture; beyond the risks they run personally, they will certainly not care about educating their children to maintain correct posture. This problem becomes even more relevant considering that the number of children is high in subjects who have a negative behavior toward maintaining a correct posture. Therefore, these results may lead to the hypothesis that an educational program would be better carried out in a population a high number of children; this can also emphasize the effect of the program, as it would be rebounded on more persons simultaneously, as it would be directed toward a family. A higher education level is statistically correlated with negative behavior and attitude toward correct posture (Table 5, Model I, II and III), even though it commonly would be expected from those with a higher education degree to have a lower tendency to correct posture since they should have a greater awareness of the risks and importance of the incorrect posture. This result can be explained because, presumably, subjects with a higher educational level hold top job positions and are consequently focused on other priorities/problems and do not pay sufficient attention to correct posture. What is very important is the result that Model III shows, that those who have high knowledge and good attitudes consequently have good behaviors regarding maintaining a correct posture. This result is coherent with a recent study, carried out by Minghelli et al., in 2021, that showed that increasing the knowledge regarding posture leads to a marked improvement in posture, although the best results were obtained in the groups that were subjected to individual correction, and the study was carried out exclusively with adolescents [39]. Also, Khalili, who analysed two groups of workers, both starting from homogenous levels of knowledge and behaviors about posture, noted that only the group subjected to a training process aimed at improving posture [40]. Synthetically, Model III shows that subjects who have behaviors not aimed at maintaining correct posture are adults, single, with multiple children and a high level of education and therefore a practical training campaign focused on improving posture should be target to adults not engaged in a relationship and with a higher level of instruction, who allegedly are occupied in more sedentary works, and as evidenced by this study have the worst posture. In Model II, it emerged that increasing knowledge determines an improvement in attitudes, and as confirmed in Model III, consequently results in behavior that maintains a correct posture. Therefore, an educational program based on the enrichment of knowledge on maintaining correct posture is necessary in adolescence before attitudes are formed [24]. All this is also confirmed by Epstein, who enlightened that in adolescents, instructed on the importance of correct posture, a better correction of posture is obtained by associating an increase in knowledge on the problem with feedback mechanisms [31]; moreover, the efficacy of educational programs has been assessed in recent studies. Minghelli (2022) showed that the influence of interventions aimed at modifying posture can be achieved by modifying incorrect postural hygiene habits [31,41,42]. Moreover, as confirmed by Model I, the prototype of a person with the worst knowledge regarding posture is a smoker and a person with a low education level who likely is less focused on a healthier lifestyle. This evidence led us to the following conclusion: to improve correct posture in the general population, it is important to develop educational programs targeted the population cohort of at smokers with a lower educational level to increase knowledge regarding the assessment of a correct posture. Finally, regarding the attitudes towards maintaining a correct posture, Model II indicates that the subjects who have negative attitudes are adults, smokers, singles, those with few children, and those with high educational qualifications. However, the aforementioned training programs towards behavior and the educational programs targeted at the enrichment of knowledge will fill the lack of attitudes towards maintaining a correct posture. In conclusion, it is important to enlighten the absence of previous reference studies in the literature regarding attitudes on maintaining correct posture, and it is necessary to conduct further studies that analyze the attitudes of the general population, from adolescence to adulthood.

## 4. Conclusions

The results of this study evidence that knowledge, attitudes and practices regarding posture are unevenly diffused in the general population and yet this topic, although it is important, is not sufficiently known. Some behaviors, that are crucial to maintaining a correct posture, although simple and efficient, are not common practices; nevertheless, most of the people comprising the sample are aware of the risks connected to a bad posture. Posture is not seen as a serious problem, and only few people contact a specialist for a consult. The most relevant discovery of this study is that the prototype of a person with the worst behaviors regarding posture is an older smoker with a low education level who is likely less focused on a healthier lifestyle. Another relevant result regards the influence of both knowledge and attitude on correct posture behaviors. These results have led us to the following conclusion: to improve correct posture in the general population, it is important to develop educational programs targeted at increasing knowledge, as well as training programs to correct postural behaviors. In conclusion, this study evidenced the importance of attitude in maintaining correct posture and the lack of references in the literature and, therefore, the importance of conducting further studies regarding attitudes of the general population.

The study was limited by the questionnaires capturing only self-reported behaviors, so that the respondents may have felt pressure to provide socially acceptable answers; however, social desirability bias may have been somewhat allayed, since the participants were assured of anonymity and confidentiality at the time of submitting the questionnaire.

## Figures and Tables

**Table 1:** Study population characteristics.

Study Population	N	Percentage
**Gender**	**1177**	
Male	598	50.8
Female	579	49.2
**Age**		
<30	423	35.9
31–35	275	23.4
36–40	150	12.7
41–45	95	8.1
46–50	45	3.8
>51	189	16.1
**Smoke**		
Smoker	324	27.5
Nonsmoker	853	72.5
**Single**		
Yes	395	33.6
No	782	66.4
Sons		
Yes	519	44.1
No	658	55.9
**How many sons?**		
0	3	0.3
1	148	12.6
2	296	25.1
3	37	3.1
4	20	1.7
5	11	0.9
No answer	662	56.2

**Table 2:** Knowledge of respondents toward the posture.

N.	Statement (Variables)	Agree (%)	Uncertain (%)	Disagree (%)
**K1**	Posture is defined as the habitual attitude of the person.	70.4	13.6	16.0
**K2**	Posture is defined as the position we have when we stand.	54.5	22.9	22.5
**K3**	Posture is defined as the position we have when we are sitting.	48.7	26.6	24.7
**K4**	Bad posture must always be corrected.	71.3	17.5	11.2
**K5**	There are courses of “postural” gymnastics.	81.1	12.9	6.0
**K6**	There are devices that improve posture.	73.4	20.3	6.3
**K7**	There are professionals who deal with posture.	80.8	13.2	6.0
**K8**	Scoliosis can be caused by poor posture.	69.8	18.3	11.9
**K9**	Neck pain cannot be caused by poor posture.	32.5	12.4	55.1
**K10**	Ergonomics studies solutions to improve posture in both living and working environments.	61.5	31.9	6.6
**K11**	As recommended by computer ergonomics, keep your head straight, not tilted up or down.	63.8	28.1	8.1
**K12**	In Italy, ergonomics in the workplace are regulated by Law 81 of 2008.	40.4	53.6	6.0

**Table 3:** Attitude of respondents toward the posture.

N.	Statement (Variables)	Agree (%)	Uncertain (%)	Disagree (%)
**A1**	The mass media speak little of posture.	62.8	32.0	5.2
**A2**	It is important to consult an expert to assess your posture.	72.6	16.0	11.4
**A3**	It is always necessary to call out those who have a bad posture.	56.4	29.7	13.8
**A4**	It is better to stay comfortable than to maintain a correct posture.	18.1	19.5	62.4
**A5**	It is more comfortable to read when lying down.	24.6	31.1	44.4
**A6**	The most relaxing way to watch a movie is by lying down.	41.4	26.8	31.9
**A7**	Proper posture in the workplace is not treated properly.	68.7	23.3	8.0
**A8**	It is difficult to maintain a good posture for a long time.	78.6	15.0	6.5
**A9**	**Children’s school backpacks should have a weight limit.**	**81.1**	**8.7**	**10.2**

**Table 4:** Behavior of respondents.

N.	Questions	Always (%)	Often (%)	Sometimes (%)	Never (%)
**B1**	Are you attentive to posture when walking or standing?	10.8	18.2	42.0	29.1
**B2**	Do you practice postural gymnastics?	4.2	1.3	14.9	79.6
**B3**	Do you go to a posturologist for joint problems?	2.8	1.4	13.8	81.9
**B4**	When you are at the computer, do you maintain a correct posture?	4.3	14.8	45.5	35.3
**B5**	Do you pay attention to posture when watching TV?	4.6	9.0	39.2	47.2
**B6**	When you buy shoes, do you choose them for their comfort?	30.0	33.9	16.8	19.3
**B7**	When you lift heavy loads, are you attentive to the posture you assume?	26.8	23.0	26.7	23.4
**B8**	Do you use a computer lift to improve your posture?	12.7	7.1	16.8	63.4
**B9**	Do you use a footrest when you are at the computer?	3.3	4.0	8.3	84.4
**B10**	Do you inquire about how to improve posture?	9.9	12.7	36.1	41.2
**B11**	Do you advise people close to you to improve their posture?	20.5	17.9	30.8	30.8
**B12**	Do you sit more than four hours during the day?	46.4	20.6	18.9	14.0
**B13**	Do you suffer from neck pain?	35.7	11.7	34.4	18.2
**B14**	Do you suffer from back pain?	29.4	15.2	40.6	14.8
**B15**	Do you suffer from sciatica?	20.8	7.6	22.3	49.2

**Table 5:** Results of the linear multiple regression.

	Coefficients not Standardized	Coefficients Standardized			
	b	Standard Error	Beta	t	*p*-Value
Model I—Dependent variable: Knowledge					
Age	−0.044	0.020	−0.118	−2.168	0.031
Gender	0.580	0.353	0.06	1.643	0.101
Smoke	3.958	0.396	0.337	10.005	0.000
Single	−0.813	0.602	−0.059	−1.352	0.177
Sons	2.698	2.196	0.042	1.229	0.220
How many children	−0.487	0.244	−0.085	−1.995	0.047
Education	2.925	0.296	0.421	9.879	0.000
**Model II—Dependent variable: Attitudes**					
Age	−0.093	0.015	−0.288	−6.393	0.000
Gender	0.173	0.250	0.021	0.689	0.491
Smoke	3.566	0.306	0.352	11.657	0.000
Single	1.394	0.426	0.118	3.272	0.001
Sons	3.956	1.554	0.072	2.545	0.011
How many children	0.344	0.173	0.069	1.984	0.048
Education	−0.499	0.228	−0.083	−2.183	0.029
KTOT	0.408	0.031	0.473	12.998	0.000
**Model III—Dependent variable: Behavior**					
Age	−0.110	0.041	−0.157	−2.66	0.008
Gender	−0.252	0.689	−0.014	−0.367	0.714
Smoke	−0.122	0.947	−0.006	−0.129	0.897
Single	3.185	1.184	0.123	2.691	0.007
Sons	−3.268	4.301	−0.027	−0.76	0.448
How many children	−1.124	0.478	−0.104	−2.35	0.019
Education	−2.586	0.631	−0.197	−4.097	0.000
KTOT	0.730	0.100	0.387	7.319	0.000
ATOT	0.573	0.122	0.262	4.686	0.000

## Data Availability

The data that support the findings of this study are available upon reasonable request from the corresponding author. The data are not publicly available due to privacy or ethical restrictions.
